# Decision support systems for antibiotic prescription in hospitals: a survey with hospital managers on factors for implementation

**DOI:** 10.1186/s12911-024-02490-7

**Published:** 2024-04-15

**Authors:** Pinar Tokgöz, Stephan Krayter, Jessica Hafner, Christoph Dockweiler

**Affiliations:** School of Life Sciences, Department Digital Health Sciences and Biomedicine, Professorship of Digital Public Health, University of Siegen, 57068 Siegen, Germany

**Keywords:** Decision support systems, Antibiotic prescription, Hospital, Artificial intelligence, Implementation

## Abstract

**Background:**

Inappropriate antimicrobial use, such as antibiotic intake in viral infections, incorrect dosing and incorrect dosing cycles, has been shown to be an important determinant of the emergence of antimicrobial resistance. Artificial intelligence-based decision support systems represent a potential solution for improving antimicrobial prescribing and containing antimicrobial resistance by supporting clinical decision-making thus optimizing antibiotic use and improving patient outcomes.

**Objective:**

The aim of this research was to examine implementation factors of artificial intelligence-based decision support systems for antibiotic prescription in hospitals from the perspective of the hospital managers, who have decision-making authority for the organization.

**Methods:**

An online survey was conducted between December 2022 and May 2023 with managers of German hospitals on factors for decision support system implementation. Survey responses were analyzed from 118 respondents through descriptive statistics.

**Results:**

Survey participants reported openness towards the use of artificial intelligence-based decision support systems for antibiotic prescription in hospitals but little self-perceived knowledge in this field. Artificial intelligence-based decision support systems appear to be a promising opportunity to improve quality of care and increase treatment safety. Along with the Human-Organization-Technology-fit model attitudes were presented. In particular, user-friendliness of the system and compatibility with existing technical structures are considered to be important for implementation. The uptake of decision support systems also depends on the ability of an organization to create a facilitating environment that helps to address the lack of user knowledge as well as trust in and skepticism towards these systems. This includes the training of user groups and support of the management level. Besides, it has been assessed to be important that potential users are open towards change and perceive an added value of the use of artificial intelligence-based decision support systems.

**Conclusion:**

The survey has revealed the perspective of hospital managers on different factors that may help to address implementation challenges for artificial intelligence-based decision support systems in antibiotic prescribing. By combining factors of user perceptions about the systems´ perceived benefits with external factors of system design requirements and contextual conditions, the findings highlight the need for a holistic implementation framework of artificial intelligence-based decision support systems.

**Supplementary Information:**

The online version contains supplementary material available at 10.1186/s12911-024-02490-7.

## Background

Antimicrobial resistance (AMR) is a major public health concern worldwide. Every year, 700.000 people worldwide die from drug-resistant infections [1]. In Germany, 54.500 people fall ill due to infections with antimicrobial-resistant pathogens each year, of which approximately 2.400 die [2]. Antibiotic prescription is an area of particular complexity within medical decision-making, as AMR is associated with high morbidity, mortality and significant healthcare expenditure [3]. The AMR crisis has been attributed, to a significant extent, to the misuse and overuse of antibiotics in both, the outpatient sector as well as care in hospitals [4]. Despite coordinated efforts and initiatives, like Antibiotic Stewardship Programs (ASP), hospitals worldwide currently face significant problems with inappropriate antimicrobial use, induced by, e.g., antibiotic intake in viral infections, incorrect dosing and incorrect dosing cycles [5, 6], with as much as 30–50% of that usage being unnecessary or inappropriate, leading to several health-related and societal consequences [7]. This suggests that current strategies and prescribing guidelines are insufficient to change practice and reduce AMR. Moreover, healthcare facilities, and particularly hospitals, are embedded in legal, economic, socio-structural, organizational, and cultural contexts that can also influence decision-making processes [8].

Decision support systems (DSSs) are computerized tools designed to support diagnostic or therapeutic decision-making to improve clinical practice and quality of care [9]. Classically, DSSs use knowledge systems that rely on if-then rules. Increasingly, machine-learning techniques are used, where large data sets are used to learn from further events and recognize specific patterns. Both methods base on artificial intelligence (AI) that combine various applications [10]. In the field of infectious diseases, AI-based DSSs have been increasingly used to assist clinicians´ decision-making in antibiotic management in hospital settings [11, 12]. They provide expert or evidence-based recommendations to promote the appropriate choice of antibiotics, dosage and treatment duration [13]. Several studies have shown many benefits of AI-based DSSs in antibiotic prescription, such as improvement in antibiotic selection, reduction in antibiotic usage, shorter length of hospital stay, decreased mortality and decreased healthcare costs [14, 15]. Despite the growing evidence in this field, there remains some level of inconsistency about the relative merits of AI-based DSSs in influencing practice patterns in hospitals, how to implement them and what refinements are needed to tailor the systems to local contexts [16]. Implementation as well as acceptance of AI-based DSSs can be challenging due to the interplay of technology, organization and user groups [17]. To analyze how these three aspects affect the implementation of DSSs, the Human, Organization and Technology-fit (HOT-fit) framework by Yusof et al. (2008) was taken into consideration [18]. With a focus directly on the healthcare industry, Yusof et al. integrated human, organizational and technological dimensions to assess the framework. Their research attempted to investigate whether or not health information systems performed as expected and to what degree they supported healthcare services. This framework includes the major domains that must be considered when adopting and implementing any technological innovation within the context of the hospital industry. These are the human, organizational and technological domains. The human domain assesses systems in terms of system use and user satisfaction. User satisfaction can be related to perceived usefulness and user acceptance towards information systems that are influenced by personal characteristics [18]. The organizational domain comprises aspects of organizational structure and organizational environment. The organizational structure consists of leadership, staff support, management, communication strategies and infrastructure. Health organizations, especially hospitals, must have the ability to prepare staff to adopt to new systems or changes that may occur [19]. The organizational environment consists of sources of funding, governance, politics and competition [18]. The technology domain consists of the system quality, information quality and service quality. The quality of system involves the linkages of features in the system including system performance and user interface. Information quality focuses in information produced and provided by the system, whereas service quality focuses on the overall support received by the system or technology service provider [18]. Criteria that can be used to assess can be gathered from Table 1.

**Table 1:** Description of the HOT-fit domains [18]

Domain	Description
**Human**	
System use	System Use relates to the person who uses it, their levels of use, training, knowledge, expectation and attitude.
User satisfaction	User satisfaction is the overall evaluation of the user’s experience in using the system. It can be related to perceived usefulness and user’s attitudes towards the systems which are influenced by user’s personal characteristics.
**Organization**	
Structure	The organizational structure consists of type, culture, politics, hierarchy, system planning and control, management and communication strategies. Leadership, support from top management and staff support are important parts of measuring the success of the system.
Environment	The organizational environment consists of sources of funding, governance, politics, competition, inter-organizational relations and communication.
**Technology**	
System Quality	The quality of the system in health care institutions involves the linkages of features in the system including system performance and user interface. Ease of use, ease of learning, response time, usefulness, availability, flexibility, and security are variables or factors that can be assessed from the quality of the system.
Information Quality	Information quality focuses on information produced by information systems including patient medical records, reports and prescriptions. Criteria that can be used to assess the quality of information include completeness, accuracy, timeliness, availability, relevance and consistency.
Service Quality	Service quality focuses on the overall support received by the system or technology service provider. Service quality can be assessed by speed of response, assurance, empathy and service follow-up
**Net Benefits**	
Net benefits capture the balance of positive and negative impacts on user, which includes clinicians, managers and IT, staff, system developers, hospitals or the entire healthcare sector. Net benefits can be assessed using direct benefits, job effects, efficiency, effectiveness, error reduction, communication, clinical outcomes and cost.	

Furthermore, the fit of the three domains is closely related to the net benefits. The net benefits comprise the positive and negative effects of a new system use and can be seen at the individual level as well as at the institutional and societal levels [18]. Validated with several studies, the HOT-fit framework is helpful for understanding relationships and alignment between the three domains as well as problems related to system performance. Although the framework has been used extensively in the evaluation of the hospital information system [19, 20] it can be beneficial to investigate human as well as technical and organizational aspects in the implementation process of other information systems. Therefore, the HOT-fit model can serve the purpose of incorporating human and organizational context-related constructs, which are essential for DSS implementation.

Consequently, the implementation and acceptance in hospitals depend on wide-ranging contextual, organizational and interpersonal determinants. From the perspective of clinicians, factors such as compatibility with existing systems, functionality and manageability of AI-based DSSs, participation of potential user groups in the planning, development and implementation phases, as well as trustworthiness of the systems, are essential for successful implementation [21, 22]. Besides, in hospital settings, decisions on investment in and implementation of new treatment options are made on the management level. Hospital managers are authorized persons who are not personally involved in a direct treatment context but who have decision-making authority within the hospital organization and whose decisions regarding the organizational processes can have an impact on the type of treatment given [23]. The joint tasks of hospital management lead to decisions that affect the interests of individual hospital departments and, at the same time, require coordination. This includes, for example, decisions on introducing new procedures in medical diagnostics and therapy, especially if additional personnel or material expenses are involved [23].

In contrast to existing studies that examine the use of AI-based DSSs from the perspective of practicing clinicians [24, 25], this study focuses on the perspective of the hospital management as the attitude of hospital managers on AI-based DSS implementation has not received adequate attention yet. Therefore, the aim of this article is to analyze implementation factors for the use of AI-based DSSs in hospitals from the management level´s viewpoint.

## Methods

### Conceptual framework and survey instrument design

We developed and piloted a survey instrument (Additional file 1) including closed questions along with the three domains of the HOT-fit model [18] and implementation factors based on the theoretical background and the review of findings in the literature as well as open-ended questions which was implemented in *Unipark* survey software.

First, the participants have been asked to prioritize problems with regard to antibiotic prescription in hospitals. Furthermore, they were asked about the existence of a DSS in the hospital they work at and an appraisal about their self- perceived state of knowledge in the context of DSSs. Based on this, the participants have then been asked to appraise implementation factors along with the three domains of the HOT-fit model as well as net benefits. The role of trust in new technologies has always been central to the acceptance and implementation of new technologies and is perhaps more important today than ever before [26]. Trust in DSSs is closely related to intention to use the system, user satisfaction, and acceptance. Furthermore, initial research showed that trust in DSSs positively affected performance and well-being, leading to more effective use of cognitive skills [27]. Consequently, trust-related questions based on the work of Gefen et al. [28], Ortega et al. [29] and Tung et al. [30] were adapted and integrated into the survey. At the end of the survey, sociodemographic aspects as well as hospital characteristics (e.g., ownership, digitization level) have been collected.

The responses to the survey questions were collected through a five-point Likert scale. After the completion of the draft survey questions, a pretest including five participants with three chief physicians and two nursing managers was conducted that aimed to assess the extent to which the survey questions reflected the domains of interest. Some amendments were suggested. The wording of the questions was subsequently modified based on the feedback from the respondents.

### Participants and data collection

People of the management level working in a hospital and having decision-making authority were included in this study. People working as hospital managers in psychiatric/geriatric or rehabilitation facilities were excluded as not to be scope of aim. To recruit participants, we conducted online research of relevant representatives of the management level in all inpatient hospitals in Germany. This search was conducted via the directory of hospitals and preventive care or rehabilitation facilities of the Federal Statistical Office. Using the general contact data of the directory, we identified the responsible person(s) of the facility (*n* = 1416). We then invited the hospital representative via email to participate in the survey or to forward the survey link to a responsible representative. The online survey was administered in German and was available from December 2022 to May 2023. We sent a monthly reminder email to all contacts to incentivize those who have not yet participated to attend the survey.

Survey data were analyzed using descriptive statistics. Since the aim of this study is to examine the perspective of hospital managers as an under-explored stakeholder group, frequency distribution of the survey questions have been obtained to identify patterns in the responses. Responses to closed-ended questions were imported into MS Excel and IBM SPSS Version 22.0 for analysis. Responses to open-ended questions were independently read by two authors (PT and SK), who separately carried out thematic analysis by identifying the core number of high-level themes for this article.

## Results

### Sample description

A total of 118 participants completed the survey and could be considered for further analysis. The sample includes 36 female (30%) and 81 male persons (69%). One person did not respond to the question about gender. Table 2 shows the demographic data of the respondents enrolled in the survey. Fifteen respondents were between 20 and 40 years old (13%), 74 people reported being between 40 and 60 years old (62%), and 26 people were older than 60 years old (22%). Three people did not provide any information on this question. Most respondents were from the clinician leadership group (61%). Sixteen persons worked as nursing managers (14%). 25% assigned themselves to other professional leadership groups, which comprised, among others, the activity of hospital pharmacy management, hospital hygiene and ASP-management. Twenty respondents (17%) had less than five years of professional experience, 32 participants (27%) had between five and ten years of professional experience, 43 of the respondents (36%) worked in the position for between eleven and 20 years, and 23 respondents (20%) reported professional experience of more than 20 years.

**Table 2:** Personal characteristics of the respondents (*n* = 118)

		n	%
**Gender**	Female	36	30%
	Male	81	69%
	No information	1	1%
**Age**	20–30 Years	2	2%
	31–40 Years	13	11%
	41–50 Years	25	21%
	51–60 Years	49	41%
	> 60 Years	26	22%
	No information	3	3%
**Professional occupation**	Medical management	72	61%
	Nursing Management	16	14%
	Other	30	25%
**Professional Experience**	< 5 Years	20	17%
	5–10 Years	32	27%
	11–20 Years	43	36%
	> 20 Years	23	20%

**Fig. 1:**
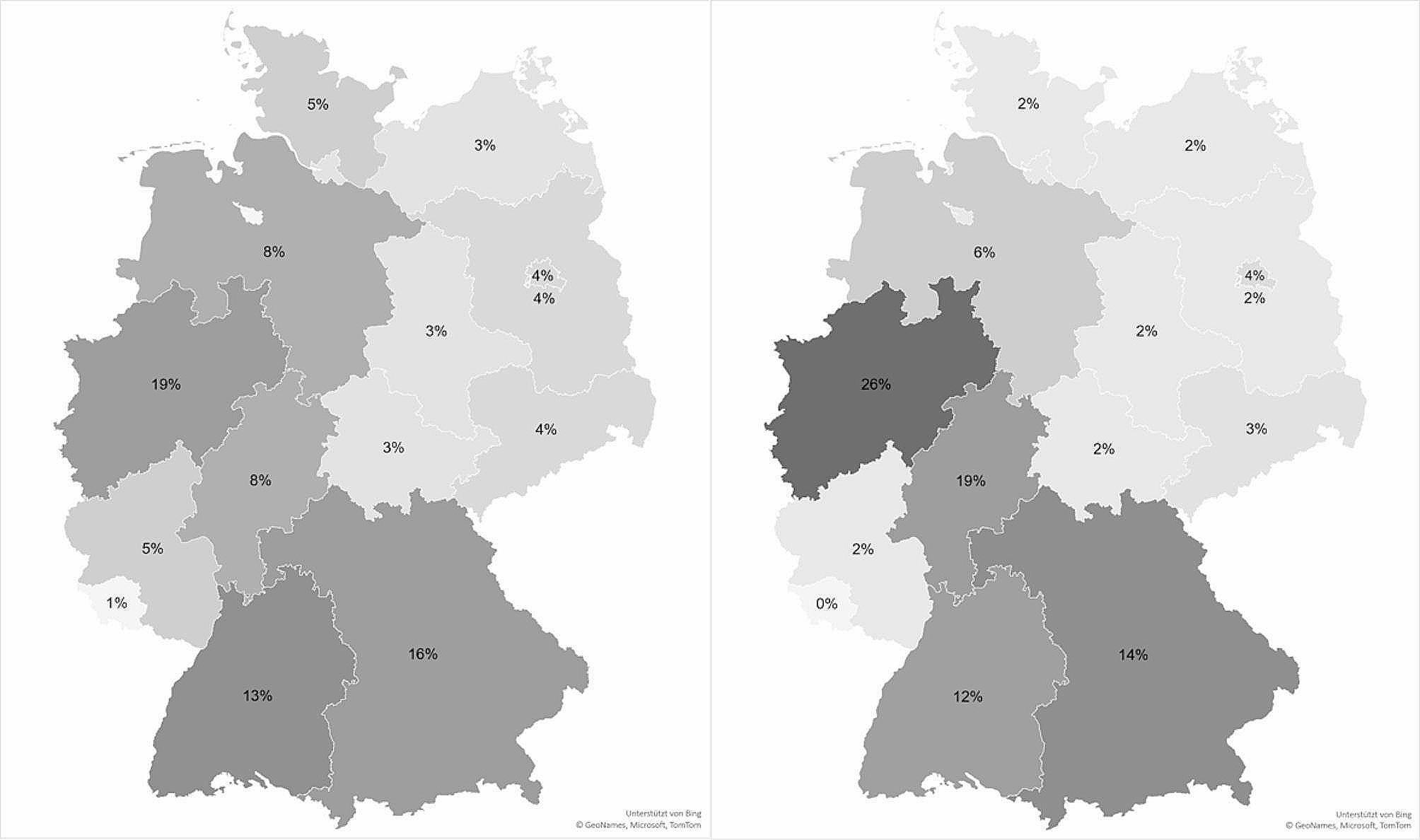
Distribution of operating hospitals in Germany (left) and hospitals participating in the survey (right) (Own representation based on data of the Federal Statistical Office, 2021 [31])

The majority of respondents were professionally active in Northrhine-Westphalia (26%), followed by the state of Hesse (19%), Bavaria (14%) and Baden-Württemberg (12%). No participating person worked in the state of Saarland. Figure 1 illustrates the distribution of hospitals in the survey in comparison to the distribution of hospitals in Germany. Our sample nearly matches the overall German distribution, with a slight overrepresentation of Northrhine-Westphalia, Rhineland-Palatinate and the state of Hesse.

#### Hospital characteristics and digitization level

The hospitals in the sample were with a percentage of 35% non-profit, 46% public and 19% private facilities. They had 151–500 planned beds in 56% of cases, and just under a third (31%) had a bed size of 501 to over 800. Smaller facilities under 150 beds were found in only 13% of cases (Table 3). When asked how digital the participants consider the hospital they work in, 23% responded with “rather digital” and 7% with “very digital”, whereas over half of the respondents (53%) described the hospital as “partly digital” and 15% as “rather not digital” and 2% “not digital at all”. Related to this question, most mentioned digital systems were hospital management systems (98%), electronic patient record (59%), or the computerized physician order entry (49%). Only 5% (*n* = 6) of the hospitals participating in the survey already used a DSS in the context of antibiotic prescribing.

**Table 3:** Hospital characteristics (*n* = 118)

		N	%
**Ownership**	Non-Profit	41	35%
	Public	55	46%
	Private	22	19%
**Planned Beds**	< 50	1	1%
	50–150	14	12%
	151–300	37	31%
	301–500	29	25%
	501–800	22	18%
	> 800	15	13%
**Digitization Level**	Not at all	2	2%
	Rather not	18	15%
	Partly	62	53%
	Rather digital	27	23%
	Very digital	9	7%
**DSS use for**	Yes	6	5%
**antibiotic**	No	110	93%
**prescription**	I cannot assess	2	2%

### Supply issues related to antibiotic prescription

**Fig. 2:**
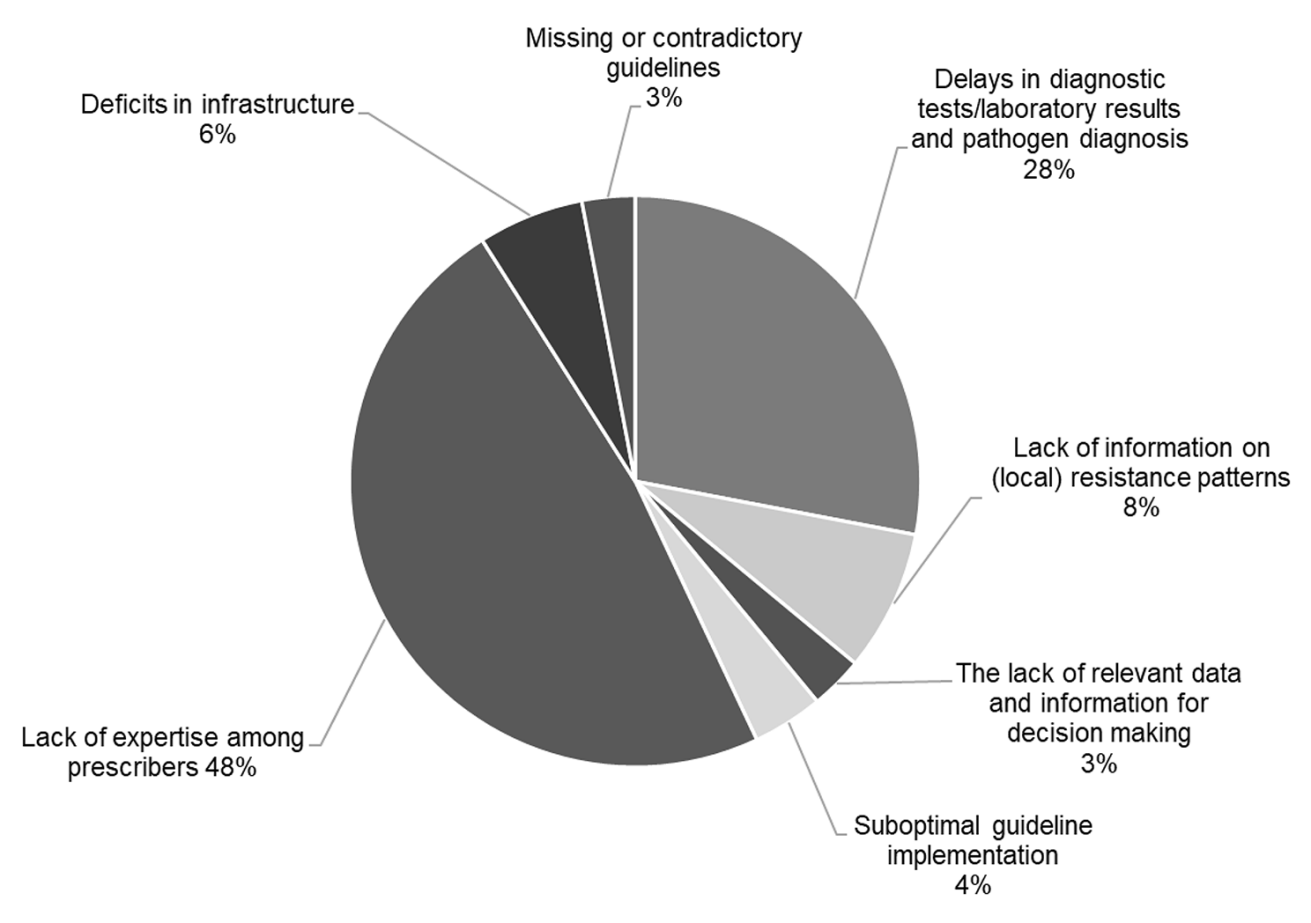
Supply issues related to antibiotic prescription in hospitals (*n* = 118)

When asked which problems regarding antibiotic prescription were present in hospitals, almost half of the respondents (48%) prioritized a lack of expertise among prescribers the most important problem (Fig. 2). Delays in diagnostic tests and laboratory results (28%) and lack of information on (local) resistance patterns (8%) were at least the top or second priority for more than a third of respondents. About 6% of the respondents assessed infrastructural deficits and 4% suboptimal guideline implementation as crucial. Respectively, 3% of the respondents ranked missing or contradictory guidelines and the lack of relevant data and information as important issues related to antibiotics prescription in hospitals.

#### Perceived benefits of AI-based DSSs for antibiotic prescription

**Fig. 3:**
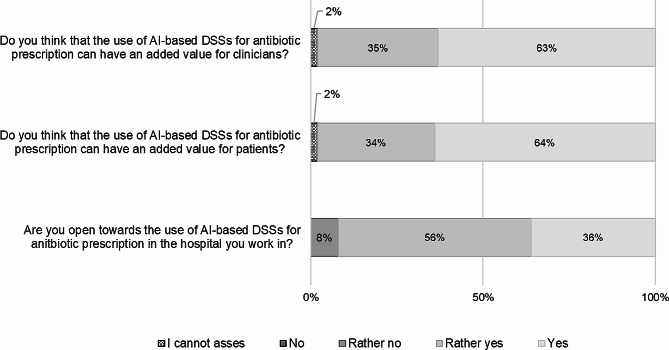
Perceived benefits of AI-based DSSs related to antibiotic prescription (*n* = 118)

As seen in Fig. 3, more than half of the respondents agreed that AI-based DSSs can have additional benefits for clinicians. 35% of people rated the statement with “rather yes”. Only two people could not give an appraisal. The situation is similar with the question of whether AI-based DSSs could have added value for patients. The majority of those questioned answered this question with “yes” (64%) or “rather yes” (34%). 92% of the people surveyed answered the question of whether they are open towards the use of AI-based DSSs for antibiotic prescription with “yes” (36%) or “rather yes” (56%). Only eight people were rather not open towards using AI-based DSSs for antibiotic prescription.

#### State of self-perceived knowledge regarding DSSs

**Fig. 4:**
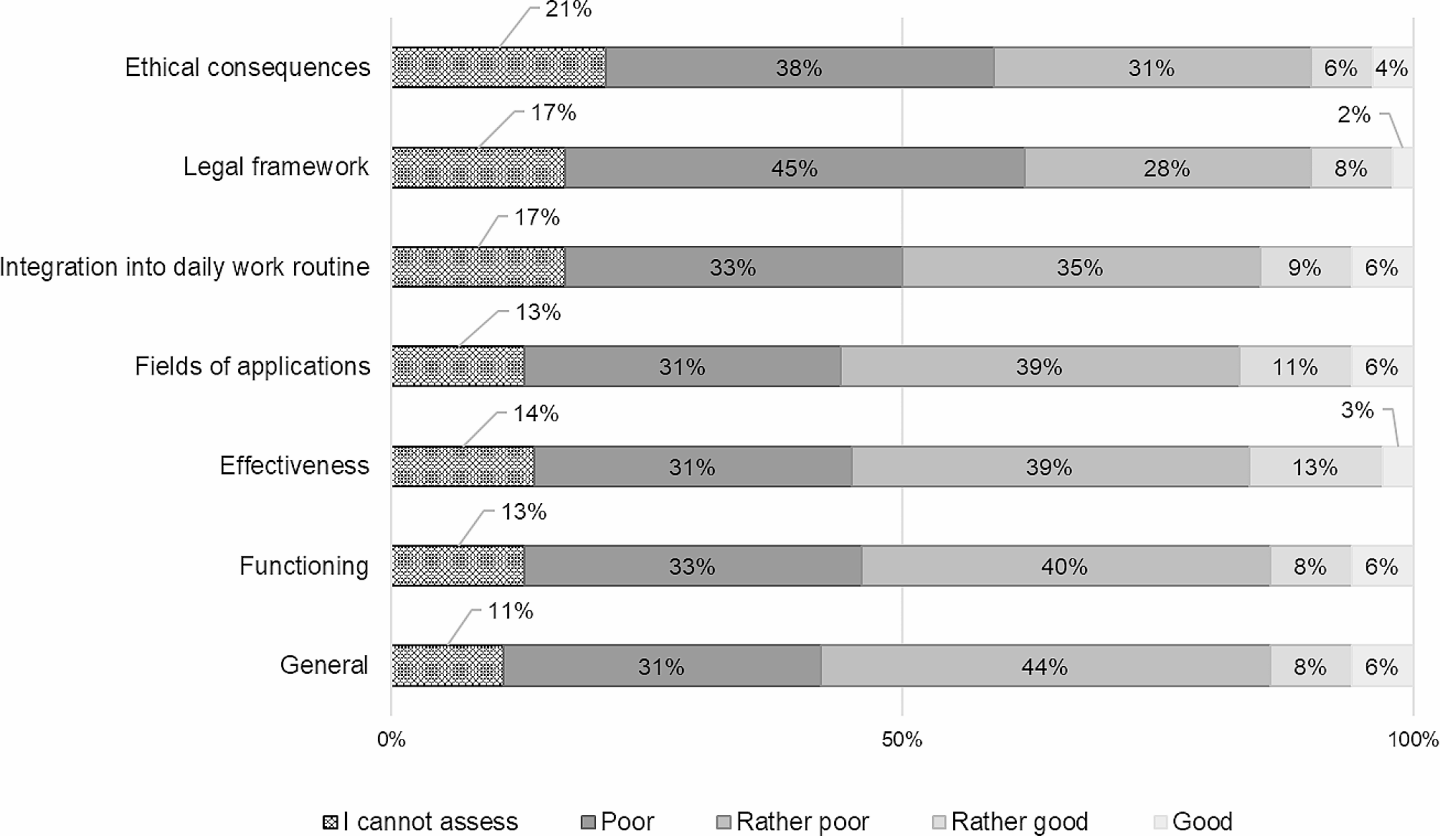
Self-perceived state of knowledge related to DSSs (*n* = 118)

It is evident that there is a gap between the perceived benefits of an AI-based DSS and its actual implementation. The possible reasons for this are also revealed by the degree of information on AI-based DSSs. Figure 4 shows that most of respondents were “rather poorly” or “poorly” informed about various aspects of AI-based DSSs. Between 68% and 75% lack essential information on functionalities, effectiveness, field of application, integration into work routine, as well as legal framework and ethical consequences. 15% of the respondents on average could not give an estimation on aspects of AI-based DSSs.

### Implementation factors

**Fig. 5:**
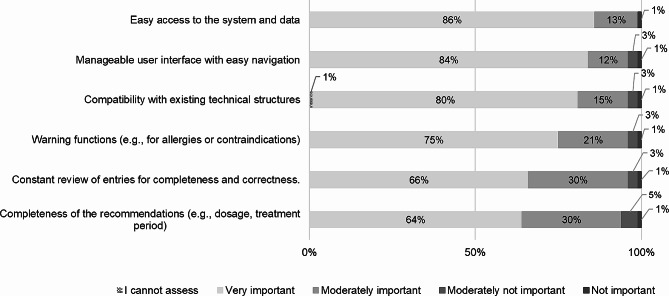
The most important technological factors for DSS implementation (*n* = 118)

Below, the implementation factors in the three domains of the HOT-fit model that had the largest percentage for being “very important” or “moderately important” are illustrated. In the context of **technological factors**, almost all respondents (99%) assessed easy access to the system and data as “very important” (86%) or “moderately important” (13%) for successful implementation. In addition, a manageable user interface with easy navigation (84% “very important” and 12% “moderately important”) and the compatibility with existing technical structures (80% “very important” and 15% “moderately important”) were assessed as the most important technological implementation factors as shown in Fig. 5. Besides, warning functions in case of allergies and/or contraindications (74% “very important” and 20% “moderately important”) as well as constant review of entries for completeness and correctness (66% “very important” and 30% “moderately important”) and completeness of the recommendations (64% “very important” or 30% “moderately important”) were rated as important factors in terms of technology. Moreover, ten people named further aspects, that were not listed. Based on the number of respondents these include, amongst others, the possibility of being able to document the analyzes carried out, the creation of negative lists with antibiotics that are explicitly not recommended and the consideration of in-house standards with regard to the antibiotics prescription.

**Fig. 6:**
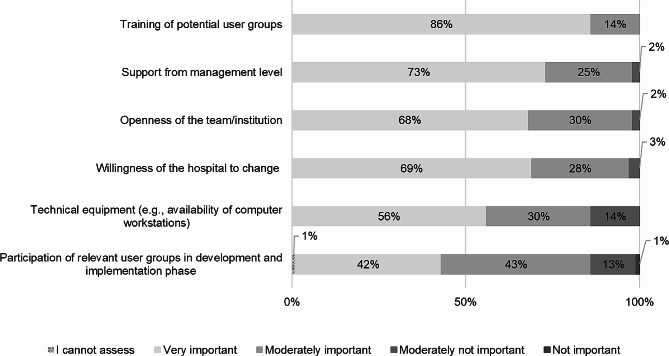
The most important organizational factors for DSS implementation (*n* = 118)

Analyzing the results on **organizational factors**, a clear tendency emerges, as Fig. 6 shows. All respondents considered the training of potential user groups to be “very important” (86%) or “moderately important” (14%) for a successful DSS implementation. In addition, 98% of the people asked, hold the opinion that the support from the management level (73% “very important” and 25% “moderately important”) and the openness of the team/institution (68% “very important” and 30% “moderately important”) as important for the implementation. Moreover, with 97%, the aspect of “hospital’s willingness to change” (69% “very important” and 28% “moderately important”) and with 96% the aspect “technical equipment” (67% “very important” and 29% “moderately important”) obtained the largest percentages in the domain of organization. Nevertheless, with almost the half of the respondents assessing as “very important” (42%) and “moderately important” (43%) the aspect of participation of relevant user groups in development and implementation phase emerges to be essential to take into account.

**Fig. 7:**
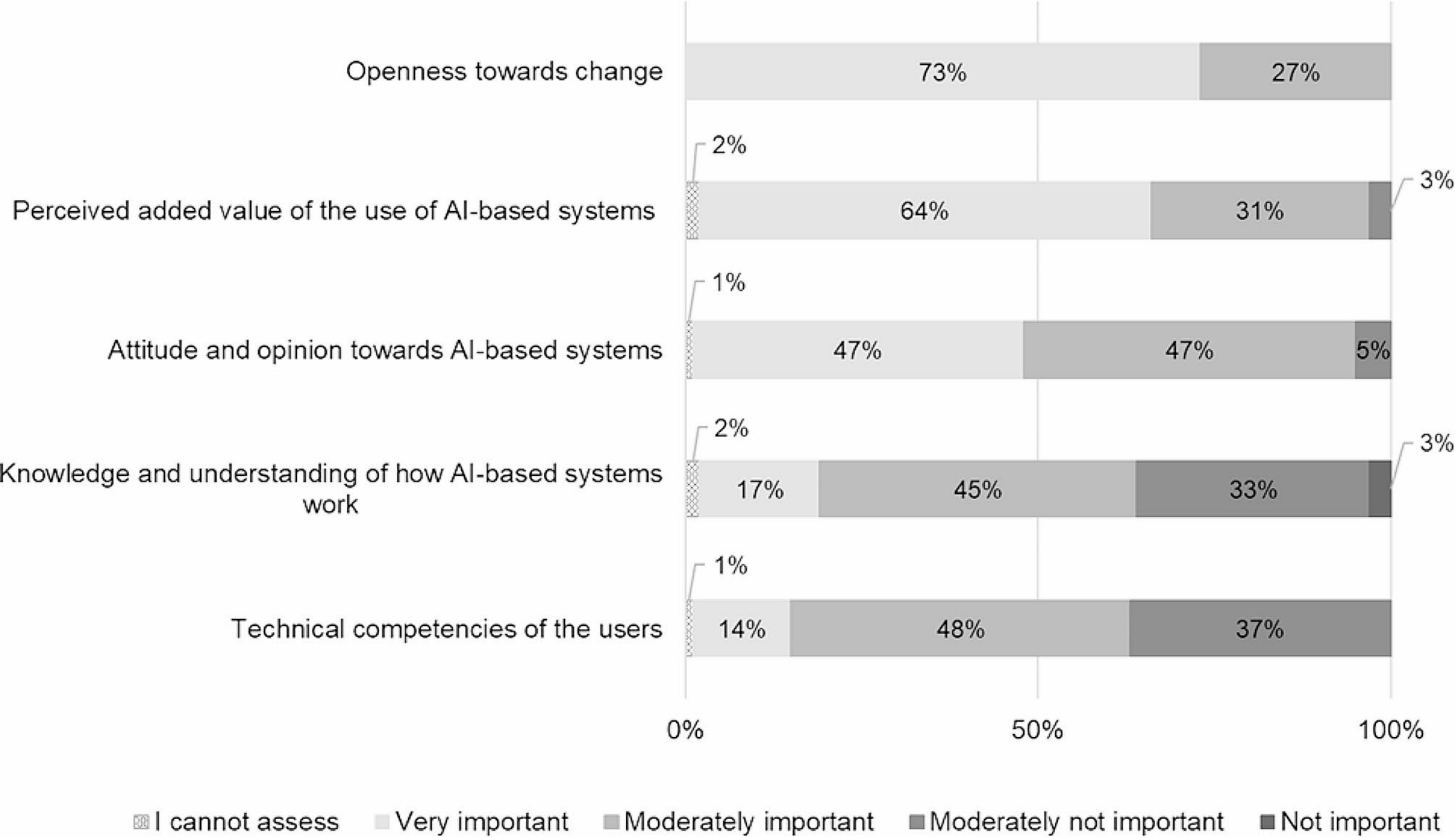
The most important human factors for DSS implementation (*n* = 118)

Considering the domain **“human”**, all of the respondents considered openness to change to be the most important factor (73% “very important” and 27% “moderately important”) for successful implementation of DSSs for antibiotic prescribing in hospitals. In addition, for 95% of those surveyed it was important (64% “very important” and 31% “moderately important”) that an added value of the use of AI-based systems is perceived, as well as for 94% (47% “very important” and 47% “moderately important”) the attitude and opinion towards AI-based systems. With 62% each, more than half of the people surveyed considered knowledge and understanding of how AI-based systems work (17% “very important” and 45% “moderately important”) as well as technical competence of the users (14% “very important” and 48% “moderately important”) as important user-related implementation factors (Fig. 7).

### Net benefits

**Fig. 8:**
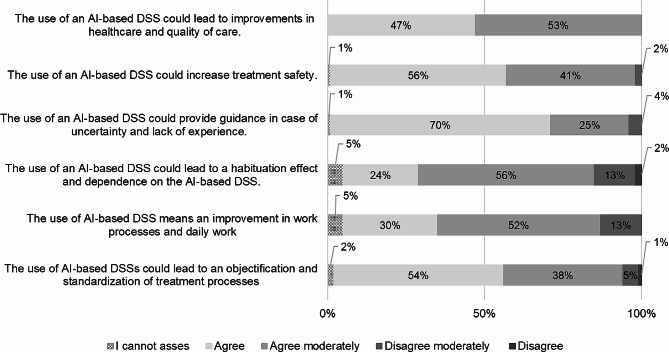
Perceived net benefits related to DSS implementation (*n* = 118)

With regard to possible impacts of the use of AI-based DSS in the context of antibiotic prescription in hospitals, there is homogenous trend, as it can be gathered in Fig. 8. Here, those factors are presented that had the largest percentage of respondents answering with “agree” or “moderately agree”. All of the respondents tended to “agree” (47%) or “agree moderately” (53%) that the use of AI-based DSSs can lead to improvements in healthcare and quality of care. Furthermore, 97% of those surveyed stated that they “agree” (56%) or “agree moderately” (41%) that the use of AI-based DSSs could increase treatment safety. For 95% of the respondents, AI-based DSSs can provide guidance in case of uncertainty and lack of experience” (70% “agree” and 25% “agree moderately”), whereby 80% of the respondents “agreed” (24%) or “agreed moderately” (56%) that the use of AI-based DSSs could lead to habituation effects and dependence on the DSS. Nevertheless, for 92% the use of an AI-based DSS could lead to an objectification and standardization of treatment processes(54% “agree” and 38% “agree moderately”) and for 82% of the respondents the use of an AI-based DSS means an improvement of work processes and daily work (20% “agree” and 52% “agree moderately”).

### Trust and trustworthiness

**Fig. 9:**
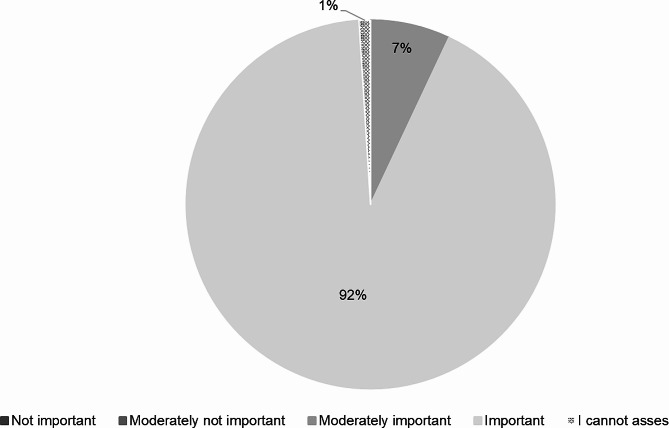
Importance of trust related to AI-based DSSs (*n* = 118)

To almost all respondents (99%) trust in an AI-based DSS was “important” (95%) or “moderately important” (7%). Only one person did not give an appraisal (Fig. 9).

**Fig. 10:**
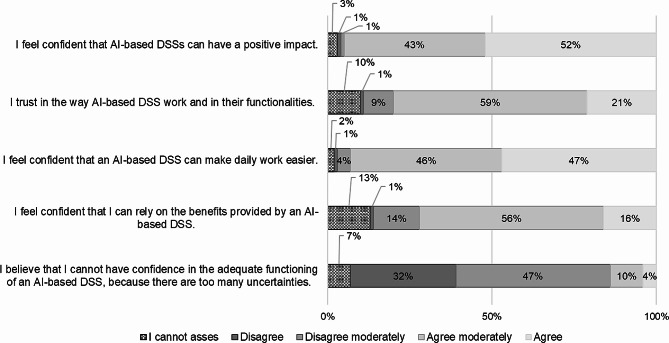
Trustworthiness of AI-based DSSs (*n* = 118)

95% of the respondents “agreed” (52%) or “agreed moderately” (43%) that they “feel confident that AI-based DSSs can have a positive impact”. In addition, 93% of them “agreed” (47%) or “agreed moderately” (46%) that they “feel confident that an AI-based DSS can make daily work easier”. 80% of the respondents rated the statement that they “trust in the way DSS work and their functionalities” with “agree” (21%) or “ agree moderately” (59%). Only 4% “agreed” and 10% “agreed moderately” to the statement that they do “believe that they cannot have confidence in the adequate functioning of an AI-based DSS, because there are too many uncertainties” (Fig. 10).

## Discussion

This research adds new knowledge to existing literature of DSS implementation by examining factors that influence its adoption in the hospital setting from the perspective of the management level as decision making authority.

### Main findings

As manifested by the identified thematic areas, the knowledge related to appropriate implementation and adoption of DSSs continues to grow. It should be emphasized that from the perspective of the management level all three dimensions of the HOT-fit model (human, organizational & technological aspects) play important roles in DSS implementation. This study contributes to the literature in several ways. First, the results emphasize the need to go beyond an approach focused on DSS attributions and its usability to consider the broader social and institutional influences that impact DSS implementation. Factors such as stakeholder groups attitudes towards using DSSs, support from management level as well as the teams´ openness and the willingness to change need to be considered when exploring the readiness of a hospital to implement DSSs. This aligns with Pope and colleagues´ suggestion that DSSs should be conceived as a computer technology and a set of practices related to this technology, which is kept in place by a network of actors in particular contexts [32]. It becomes clear that implementation in a hospital is not a completely endogenous process, rather structure of the social system and certain roles can influence the implementation of innovations. Insufficient willingness to change is often rooted in situational factors such as a lack of need or the perceived complexity of the new technology being too high [33]. Users still need to be able to take oversight of the overall process, which increasingly involves suggestions from AI-based systems.

Furthermore, the results reveal that a fundamental trust exists, but also uncertainties are present in terms of the DSS implementation and related consequences, which might be caused by a lack of knowledge. Here it is worth mentioning, that even though the respondents´ self-perceived knowledge towards AI-based DSS is rather poor, they assess users knowledge and understanding of how AI-based DSSs work to be an important factor for successful implementation. Although it might seem paradoxical at first sight, it is not mutually exclusive assessing knowledge towards DSSs to be important even when the asked person do not have enough of it, yet. Existing studies also confirm that the lack of knowledge of a new technology and the reason for its use may hinder implementation, emphasizing the importance of a suitable introduction of the system to the target group [34, 35]. In the case of DSSs, the study also shows that specifically, a crucial step is to clarify the legal framework underpinning its implementation and to offer guidance and support on how to manage with difficulties and problems. Organizational studies [34, 36] suggest that management commitment to new technologies and the reduction of uncertainties about why they are needed are key predictors of successful implementation as revealed in this work. As a result, representatives of the management level should raise awareness of the actual DSS functions and its benefits for the daily work routine. Making substantial investments in hospitals where resistance and hesitation are prominent may not be efficient nor cost-effective. Additionally, hospital managers may appeal to the positive attitudes shown by some physicians to overcome the skepticism of others.

Like explored in this work, involving physicians as a relevant user group in the developing and implementation phase for ensuring transparency and participation are considered as a key element of successful implementation. Literature also emphasizes the importance of giving project management roles to physicians [37]. Involvement of physicians in decision-making process regarding DSS implementation includes participation in the planning, development and the actual implementation phase. When physicians perceive themselves as active stakeholders, they become more willing to change their traditional work routines by using DSSs [37]. Thus, the DSS implementation should follow a user-centered approach, modulating the relationship between clinicians and rules at the micro-level. Rigid top-down regulations established at an institutional level that do not address users´ needs and preferences may cause resistance and lead to less acceptance of DSS implementation. Besides, efforts should be directed at increasing the systems´ manageability and compatibility with existing structures and workflow to overcome the barrier of implementation, which may derive from a lack of DSS integration into daily work routines [35].

All in all, the results suggest that from the perspective of hospital managers, following aspects need to be addressed when implementing DSSs for antibiotic prescription in hospitals. First, the hospital administrators should realize a strong need for continued motivation and training for physicians. This is strongly linked to the second aspect that more attention has to be paid to physicians participation in the planning, development and implementation phase of an AI-based DSS. This study suggests that physicians should actively participate in the decision-making process. Third, the DSS must have easy and manageable features as well as include user-friendly elements so that using the instructions given by the developer and the system is easy and will help to attain gains in work performance.

### Strength and limitations

This research has extended the understanding of implementation by investigating the phenomenon from a new perspective. Management-level implementation factors have never been analyzed in Germany and very little research has been conducted in similar countries [21, 38]. So, this study contributes to the existing body of knowledge by improving the current understanding of DSS implementation, which can support stakeholders in understanding the implementation process. This survey elaborates on key themes in research involving AI-based DSSs derived from the existing body of knowledge [21, 22].

The main limitation of this research is the small sample size and the participants mostly being from the clinicians´ leading group. So, the findings of this research have limitations in terms of generalization. In this survey, 5% of the respondents worked in an organization where an AI-based DSS is in use. Accordingly, assessments of possible factors for successful implementation may differ from those in whose workplace such a system is not in use, yet, so that results should be interpreted against this background. Accompanying, it would be suggested to assess and differentiate the different influences of those factors between adopters and nonadopters. Hence, these will allow more generalization of the findings.

As the objective of this survey was to describe the attitudes towards DSS implementation and identify patterns from the perspective of hospitals managers, no hypothesis testing or inference statistical analysis were conducted nor relative importance of the implementation factors assessed. It also has to be taken into account when interpreting the results, that the level of knowledge is perceived to be low, but the respondents unanimously considered AI-based DSSs to be beneficial for improving quality of care. Even though the validity of the questionnaire has not been statistically tested, literature was involved and cognitive pre-tests were conducted to assess the comprehensibility of the items and response options. Although the self-perceived level of knowledge of the participants regarding AI-based DSS was poor, it was possible to ensure that all respondents referred to this in their assessments by using a definition at the beginning of the survey.

The consideration of the HOT-fit model was expedient to systemize the implementation factors for the survey and to highlight the importance of paying attention to technical components and the users as well as the surrounding environment for the implementation process all together. Additionally, the framework may serve as a starting point for further research and testing hypothesis of successful implementation. Further studies with greater sample sizes will be the future extension towards the generalization of this study. Additionally, for a multi-perspective approach the consideration of further stakeholder groups, e.g., patients, could have an added value in terms of successful implementation.

### Implications for research, practice and policy

This study offers insights into AI-based DSS implementation from hospital managers perspective and the results might be reference points for further multi-perspective research as well as for practitioners, developers and regulators. Most of the respondents are open but have not yet the possibility to implement DSSs in their hospital. Based on the results, the following implications can be drawn.

For practitioners developers and regulators, this study highlights key factors affecting implementation of AI-based DSSs. An efficient health interoperability ecosystem provides an infrastructure that uses standards, policies and protocols to enable seamless and secure capture and utilization of health information. Additionally, the organization needs to improve the technological aspects especially those related to the accuracy of a DSS that appropriate to the needs of the hospital department. The findings of this work might provide guidance to hospital administration level selecting as well as preparing the most appropriate way and strategies of implementing DSS into hospitals. It may be advantageous for hospital administrators to implement policies for development and implementation of AI-based DSS aimed at increasing acceptance and adoption. Our findings suggest that involving potential user groups, respectively clinicians in system development, allowing user-centered explanations for AI-based DSS and educating clinicians on AI-based DSSs may be effective policies for increasing knowledge and implementation. Last, to support user´s competencies and ability there needs to be a guidebook, so it can be referred to when the users feels insecure. For academia and from a theoretical point of view, the findings can be a starting point to help in understanding AI-based DSS implementation. Future studies can extend this study based on its findings. Moreover, this study does not address how change or implementation takes place or the causal mechanism leading to adoption or non-adoption of AI-based DSSs. The implementation of AI-based DSSs should also be examined longitudinally to analyze its long-term professional and organizational effects. The findings of this work may be used for further research to assess attitudes towards DSSs more extensively across contexts and countries as well as according to differentiating structural characteristics of inpatient care. e.g., care zone or region. Finally, this type of research needs to be implemented in other system circumstances and infrastructures. Healthcare industry may vary across different countries. Therefore, future research should make cross-country comparisons to enhance the completeness of this study.

## Conclusion

This research has extended the understanding of AI-based DSS implementation and highlighted hospital managers perspective related to factors influencing AI-based DSS implementation. The organizational environment along with user´s perception are crucial for DSS implementation. The effective diffusion of DSSs demands effective re-orientation of hospitals to establish a supportive and facilitating environment for the uptake of DSSs. Therefore, setting dynamics and user-specific requirements need to be considered to improve AI-based DSS implementation and its use for antibiotic prescription in hospitals.

### Electronic supplementary material

Below is the link to the electronic supplementary material.

Supplementary Material 1

## Data Availability

The data that support the findings of this study are available from the corresponding author upon reasonable request.

## Abbreviations

AI: Artificial Intelligence
AMR: Antimicrobial resistance
ASP: Antibiotic Stewardship Program
DSSs: Decision Support Systems
HOT-fit-model: Human-Organization-Technology-fit-model
