# Supplementary material for: Decision support systems for antibiotic prescription in hospitals: a survey with hospital managers on factors for implementation
Source: BMC Med Inform Decis Mak. 2024 Apr 15;24:96. doi: 10.1186/s12911-024-02490-7 (PMC11020884; doi:10.1186/s12911-024-02490-7)
Supplement: Supplementary file 1 — Supplementary Material 1 [file 12911_2024_2490_MOESM1_ESM.docx]

Additional file 1: Survey questionnaire

**1. Antibiotic resistance is a global challenge. In order to prevent the emergence of antibiotic resistance, it is essential to prescribe antibiotics according to need. Please prioritize the following problems that, in your view, counteract adequate antibiotic prescribing. The most important problem should be at the top, the least important problem at the bottom.**

- Delays in diagnostic tests/laboratory results and pathogen diagnostics

- Lack of information on (local) resistance patterns

- Missing or contradictory guidelines

- Suboptimal guideline implementation

- Lack of expertise among prescribers

- Deficits in infrastructure

- The lack of relevant data and information for decision making

1.

2.

3.

4.

5.

6.

7.

**2. If there is another important issue that you feel has not been listed, please feel free to add it here.**

________________________________________________________________________

**3. Does the hospital where you work in use an AI-based decision support system (DSS) in the context of antibiotic prescribing?**

Yes Ο

No Ο

I cannot asses Ο

**3.1 And if yes, how long has the system been in use?**

It is still in the implementation phase Ο

Less than 2 years Ο

2 to 5 years Ο

More than 5 years Ο

I cannot asses Ο

**4. How well informed do you feel regarding the aspects of AI-based DSSs for prescribing antibiotics mentioned below?**

Poor Rather Rather Good I cannot

poor good assess

General Ο Ο Ο Ο Ο

Functioning Ο Ο Ο Ο Ο

Effectiveness Ο Ο Ο Ο Ο

Fields of application Ο Ο Ο Ο Ο

Integration into daily work routine Ο Ο Ο Ο Ο

Legal framework Ο Ο Ο Ο Ο

Ethical consequences Ο Ο Ο Ο Ο

Other: Ο Ο Ο Ο Ο

**5. Below are possible attributes of AI-based DSSs for antibiotic prescribing listed. Which of the following features do you think an AI-based DSS should have?**

Disagree Moderately Moderately Agree I cannot

disagree agree assess

AI-based DSSs might help

to select the optimal and Ο Ο Ο Ο Ο

adequate antibiotic

therapy

AI-based DSSs might

provide generally accepted Ο Ο Ο Ο Ο

guideline information on

antibiotic prescription

AI-based DSSs might

provide alerts for Ο Ο Ο Ο Ο

allergies and/or

contraindications

AI-based DSSs might

provide dosage Ο Ο Ο Ο Ο

recommendation for

antibiotic therapy

Further attributes: Ο Ο Ο Ο Ο

**6. An AI-based DSS can have a wide range of technology-related characteristics. Please indicate which of the aspects presented below you consider important for a successful implementation.**

Not Moderately not Moderately Very I cannot

important important important important assess

Compatibility with

existing technical Ο Ο Ο Ο Ο

structures

Easy manual

data entry Ο Ο Ο Ο Ο

Easy access to

the system and Ο Ο Ο Ο Ο

data

Manageable user

interface with Ο Ο Ο Ο Ο

easy navigation

Clear presentation

of the results (e.g., Ο Ο Ο Ο Ο

use of tables, color

highlighting)

Automated data

transmission Ο Ο Ο Ο Ο

Warning functions

(e.g., for allergies Ο Ο Ο Ο Ο

or contraindications)

Notifications on updated

data e.g., laboratory Ο Ο Ο Ο Ο

data or changes in

guidelines

The presence of

alternative Ο Ο Ο Ο Ο

recommandations;

multiple selections

Constant review of

entries for Ο Ο Ο Ο Ο

completeness and

correctness

Providing data

security Ο Ο Ο Ο Ο

Comprehensibility of

recommendations;

information on data Ο Ο Ο Ο Ο

sources

Completeness of the

recommendations

(e.g., dosage, Ο Ο Ο Ο Ο

treatment period)

Precise

recommendation

without much room Ο Ο Ο Ο Ο

for interpretation

Consideration of

individual and/or

context-specific Ο Ο Ο Ο Ο

circumstances

Other:_________ Ο Ο Ο Ο Ο

**7. Below organizational factors for implementing AI-based DSSs are listed. Please indicate which of these factors you consider important for a successful implementation.**

Not Moderately not Moderately Very I cannot

important important important important assess

Training of

potential user Ο Ο Ο Ο Ο

groups

Technical equipment

(e.g., availability of Ο Ο Ο Ο Ο

computer workstations)

Access via mobile

devices (e.g. tablets) Ο Ο Ο Ο Ο

(Technical) support

for questions Ο Ο Ο Ο Ο

and malfunction

Participation of

relevant user

groups in Ο Ο Ο Ο Ο

development and

implementation phase

Willingness of the

hospital to change Ο Ο Ο Ο Ο

Openness of the

team/ institution Ο Ο Ο Ο Ο

Financial incentives

for the hospital to Ο Ο Ο Ο Ο

use AI-based DSSs

Redesigning and

restructuring of Ο Ο Ο Ο Ο

medical education

Funding support for

the acquisition of Ο Ο Ο Ο Ο

AI-based DSSs

Clarification of the

legal framework for

the use of AI-based Ο Ο Ο Ο Ο

DSSs (e.g., liability

issues)

Support from

management level Ο Ο Ο Ο Ο

Restructuring of

“traditional” work

processes and Ο Ο Ο Ο Ο

standards in hospitals

Other:_________ Ο Ο Ο Ο Ο

**8. Below you can see different user-related factors for the implementation of AI-based DSSs. How would you assess the importance of these factors for a successful implementation?**

Not Moderately not Moderately Very I cannot

important important important important assess

Attitude and opinion

towards AI-based

systems Ο Ο Ο Ο Ο

Previous experience

with AI-based systems Ο Ο Ο Ο Ο

Knowledge and under-

standing of how Ο Ο Ο Ο Ο

AI-based systems

work

Openness towards

change Ο Ο Ο Ο Ο

Technical competencies

of the users Ο Ο Ο Ο Ο

Professional experience

of the users Ο Ο Ο Ο Ο

Age of the users Ο Ο Ο Ο Ο

Perceived added value

of the use of AI-based Ο Ο Ο Ο Ο

systems

Other:_________ Ο Ο Ο Ο Ο

**9. How important is the trustworthiness of an AI-based DSS to you?**

Not important Ο

Moderately not important Ο

Moderately important Ο

Important Ο

I cannot assess Ο

**10. How would you assess the following statements regarding the trustworthiness of AI-based DSSs?**

Disagree Disagree Agree Agree I cannot

moderately moderately asses

I feel confident that AI-

based DSSs can have Ο Ο Ο Ο Ο

a positive impact

I trust in the way AI-

based DSS work and Ο Ο Ο Ο Ο

in their functionalities

I feel confident that an

AI-based DSS can make Ο Ο Ο Ο Ο

daily work easier

I feel confident that

I can rely on the

benefits provided by

an AI-based DSS Ο Ο Ο Ο Ο

I believe that I cannot

have confidence in the

adequate functioning of

an AI-based DSS, Ο Ο Ο Ο Ο

because there are too

many uncertainties

**11. Do you think that the use of AI-based DSSs for antibiotic prescription can have an added value for clinicians?**

No Ο

Rather no Ο

Rather yes Ο

Yes Ο

I cannot assess Ο

**12. Do you think that the use of AI-based DSSs for antibiotic prescription can have an added value for patients?**

No Ο

Rather no Ο

Rather yes Ο

Yes Ο

I cannot assess Ο

**13. Are you open towards the use of AI-based DSSs for antibiotic prescription in the hospital you work in?**

No Ο

Rather no Ο

Rather yes Ο

Yes Ο

I cannot assess Ο

**14. Hereinafter potential positive impacts of implementing and using AI-based DSSs for antibiotic prescribing are presented. How would you assess these factors in terms of successful implementation?**

Disagree Disagree Agree Agree I cannot

moderately moderately asses

Use of AI-based

DSSs could lead

to improvements Ο Ο Ο Ο Ο

in healthcare and

quality of care

The use of an

AI-based DSS

could increase Ο Ο Ο Ο Ο

treatment safety

The use of AI-based

DSSs could lead to

an objectification Ο Ο Ο Ο Ο

and standardization

of treatment processes

The use of an AI-based

DSS could provide

guidance in case of Ο Ο Ο Ο Ο

uncertainty and lack

of experience

The use of AI-based

DSS means an

improvement in work Ο Ο Ο Ο Ο

processes and daily

work

The use of AI-based

DSSs leads to time Ο Ο Ο Ο Ο

savings

The use of AI-based

DSSs means less

dependence on other Ο Ο Ο Ο Ο

professions (e.g.,

pharmacists)

The use of AI-based

DSSs could lead to Ο Ο Ο Ο Ο

cost efficiency

The use of AI-based

DSSs could contain Ο Ο Ο Ο Ο

the development of

antimicrobial resistance

Other:_____________ Ο Ο Ο Ο Ο

**15. Hereinafter potential negative impacts of implementing and using AI-based DSSs for antibiotic prescribing are presented. How would you assess these factors in terms of successful implementation?**

Disagree Disagree Agree Agree I cannot

moderately moderately asses

The use of AI-based

DSSs implies a

change in work Ο Ο Ο Ο Ο

processes and

hinders the workflow

The use of an

AI-based DSS is Ο Ο Ο Ο Ο

more time consuming

The use of AI-based

DSSs could lead to a

habituation effect and Ο Ο Ο Ο Ο

dependence on the

AI-based DSS

The use of AI-based

DSSs means an

infringement of Ο Ο Ο Ο Ο

clinicians´

professional autonomy

The use of an AI-based

DSS could have a

negative impact on the

professional role of Ο Ο Ο Ο Ο

clinicians and reinforce

“checklist”-working

The use of AI-based

DSSs could lead to Ο Ο Ο Ο Ο

more incorrect

prescriptions

The use of AI-based

DSSs leads to

transferring decision- Ο Ο Ο Ο Ο

making authority and

responsibility

The use of AI-based

DSSs could reduce Ο Ο Ο Ο Ο

quality of care

Other:______________ Ο Ο Ο Ο Ο

**16. Are there any other important aspects that were not addressed in the questionnaire and that you would like to add?**

_______________________________________________________________________

_______________________________________________________________________

**Additional Information**

**17. In which field are you professionally active?**

Medical management Ο

Nursing management Ο

Commercial management Ο

Directorial management Ο

Other:____________________________

**18. How many years have you been working in this position?**

Less than 5 years Ο

5 to 10 years Ο

11 to 20 years Ο

More than 20 years Ο

**19. In which state do you practice your profession?**

Baden-Württemberg Ο

Bavaria Ο

Berlin Ο

Brandenburg Ο

Bremen Ο

Hamburg Ο

Hesse Ο

Mecklenburg Western Pomerania Ο

Lower Saxony Ο

Northrhine-Westphalia Ο

Rhineland-Palatinate Ο

Saarland Ο

Saxony Ο

Saxony-Anhalt Ο

Schleswig Holstein Ο

Thuringia Ο

**20. What type of ownership operates the hospital where you work?**

Non-profit Ο

Public Ο

Privat Ο

**21. How many beds does the hospital have where you work?**

Less than 50 Ο

50 to 150 Ο

151 to 300 Ο

301 to 500 Ο

501 to 800 Ο

More than 800 Ο

**22. Which of the following digital systems does the hospital where you work have?**

Hospital management system Ο

Electronic patient record Ο

Computerized physician order entry Ο

AI-based DSS Ο

Other:____________________________

**23. Please give an assessment of the level of digitization in the hospital where you work.**

Not Rather Partly Rather Very

digital not digital digital digital digital

How digital would

you assess the Ο Ο Ο Ο Ο

hospital you work in?

How digital do you

think the hospital Ο Ο Ο Ο Ο

you work in should

be?

**Sociodemographic information**

**24. What age group do you belong to?**

Younger than 20 years Ο

20 to 30 years Ο

31 to 40 years Ο

41 to 50 years Ο

51 to 60 years Ο

Older than 60 years Ο

I do not want to answer this question Ο

**25. What gender are you?**

Male Ο

Female Ο

Divers Ο

I do not want to answer this question Ο
